# Autochthonous *Blastomyces dermatitidis*, India

**DOI:** 10.3201/eid3012.240830

**Published:** 2024-12

**Authors:** Anuradha Chowdhary, Gaston I. Jofre, Ashutosh Singh, Andrius J. Dagilis, Victoria E. Sepúlveda, Allison T McClure, Daniel R. Matute

**Affiliations:** University of Delhi, New Delhi, India (A. Chowdhary, A. Singh); University of North Carolina, Chapel Hill, North Carolina, USA (G.I. Jofre, V.E. Sepúlveda, A.T. McClure, D.R. Matute); Virginia Commonwealth University, Richmond, Virginia, USA (G.I. Jofre); University of Connecticut, Storrs, Connecticut, USA (A.J. Dagilis)

**Keywords:** blastomycosis, *Blastomyces*, *Blastomyces dermatitidis*, fungal infections, fungi, India, phylogenetics

## Abstract

*Blastomyces* spp. fungi, the causal agent of blastomycosis, are common in North America but do occur in other areas of the world. The most prevalent pathogen in the genus is *B. dermatitidis*. Most* B. dermatitidis* isolates originate from North America, but there are sporadic reports of *B. dermatitidis* recovery from Africa and Asia. High-quality reports that incorporate genetic information about the fungus outside North America have been rare. Genome sequencing of 3 fungal isolates from patients in India with chronic respiratory diseases revealed that the isolates belong to a genetically differentiated lineage of *B. dermatitidis*. Because the patients had no history of traveling outside of Asia, blastomycosis was most likely autochthonously acquired, which suggests a local population of *B. dermatitidis*. Our results suggest the endemic range of *B. dermatitidis* is larger than previously thought, calling for a reassessment of the geographic range of different agents of endemic mycoses.

Blastomycosis is a fungal disease prevalent among immunosuppressed patients (*1*–*5*) that can develop as a progressive disease with either pulmonary or extrapulmonary involvement (*1*–*3*,*6*–*10*). Blastomycosis is caused by *Blastomyces* spp., which are thermal dimorphic fungi with a saprophytic mycelial form in the soil and a pathogenic yeast form in hosts (*2*,*3*,*6*,*11*,*12*). The transition between those 2 forms is mediated by temperature; at ≈37°C, the mycelial (or conidia) form transforms into a yeast (*13*–*15*). Most often, human infections occur after breathing in fungal spores from the environment (*16*), but a very small fraction of cases have demonstrated sexual transmission from patient to patient (*17*,*18*).

*B. dermatitidis* is the most heavily studied species of *Blastomyces* (*4*,*5*,*19*–*21*) and is considered endemic to North America. Some reports have suggested the range of *B. dermatitidis* might extend outside North America (*22*,*22*–*25*), but the actual range of the species is debated (*23*,*26*). Reports of blastomycosis in Asia have been considered either of poor-quality diagnosis or imported (*25*,*26*). In addition, the identity of the causative agent of blastomycosis in those locales remains largely unknown. PCR-based assays suggest some of the isolates recovered from patients in India could be *B. dermatitidis* (*27*), but those reports predate the acknowledgment of the existence of multiple species of *Blastomyces*.

In this article, we report a cluster of cases of blastomycosis from India. By using genomewide sequencing and phylogenetic reconstruction, we determined the etiologic agent of blastomycosis in the 3 cases is *B. dermatitidis*. The *B. dermatitidis* isolates form a differentiated lineage from the isolates in North America. The 3 cases reported in this article have no patient history of travel outside of India, which suggest either secondary infections from a traveler (unlikely given the known mode of transmission of *Blastomyces*) or locally acquired infections.

## Methods

### *Blastomyces*-Like Isolates from India

#### Isolate Collection and Morphology

We obtained isolates from 3 patients who had chronic respiratory disease during 2006–2010 and were referred to the Medical Mycology laboratory, Vallabhbhai Patel Chest Institute, at the University of Delhi (Delhi, India) for identification of mycotic etiology. The 3 patients’ infections were unresponsive to antitubercular therapy. A detailed assessment of the patients’ travel history was taken; none reported any travel outside of India. No follow-up record is available for the patients.

We processed the samples by using standard protocols; they were anonymized before they were received. The isolates were cultured from lung aspirate, lymph node biopsy, and discharging sinus of the sternum (Appendix Table 1). We subcultured the isolates on Sabouraud dextrose agar (SDA) at 25°C to induce mycelial growth. To assess whether the isolates showed transition to a yeast form when exposed to 37°C, we inoculated conidia on pea seed agar and incubated the isolates for 7 days. We microscopically examined the isolates after mounting them with lactophenol cotton blue.

#### Genome Sequencing 

Next, we used the cultures to extract DNA from each isolate as previously described (*28*,*29*). We pooled the resulting genomic libraries sequenced in a single S4 flow cell (2 × 150 bp read length) by using the NovaSeq 6000 (Illumina, https://www.illumina.com). The target sequencing depth was 40 (Appendix Table 2). This sequencing process was done to obtain whole genome sequences of each isolate, which enabled us to differentiate the isolates from India in comparison with isolates from North America. We have described all variant calling protocols, phylogenetic, and population genetic approaches in detail in the appendix (Appendix). We deposited all analytical code related to this study into GitHub (https://github.com/gjofre/BlastomycesIndia).

## Results

**Figure 1:**
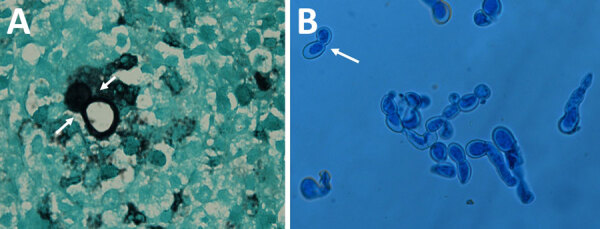
Autochthonous *Blastomyces dermatitidis* biopsy and culture findings from a patient in India. Microscopic characteristics are consistent with *Blastomyces dermatitidis*. A) Gomori’s methenamine silver–stained section of a subcutaneous nodule biopsy obtained from sternum region showing a large, thick-walled broad-based budding yeast cell (white arrows) typical of *Blastomyces*. B) Lactophenol cotton blue mount of the 6-day-old yeast form on pea seed agar at 37°C, showing numerous large thick-walled and broad-based budding yeast cells (white arrow). Original magnification is ×100.

A histological section of 1 of the samples (subcutaneous, nodule sternum) revealed the presence of a large thick-walled broad-based budding yeast with a double refractile wall (Figure 1, panel A). This cell yeast morphology is typical of *Blastomyces*. The macroscopic mycelial colony morphologies were also consistent with that of *Blastomyces*. When grown on SDA at 25°C, all 3 isolates grew white downy colonies. We conducted microscopic examination of lactophenol cotton blue mounts from SDA slide cultures of each strain that revealed thin septate hyphae, bearing spherical to pyriform smooth-walled microconidia. Subcultured colonies on pea seed agar incubated at 37°C showed conversion from a mold to a yeast form. Finally, microscopic yeast morphology in culture was also consistent with that of *Blastomyces*. Similar to the yeast cells observed in the biopsy (Figure 1, panel A), microscopic examination of the growth of yeast revealed numerous thick-walled and broad-based budding yeast cells (Figure 1, panel B), which are typical of the genus (*30*).

**Figure 2:**
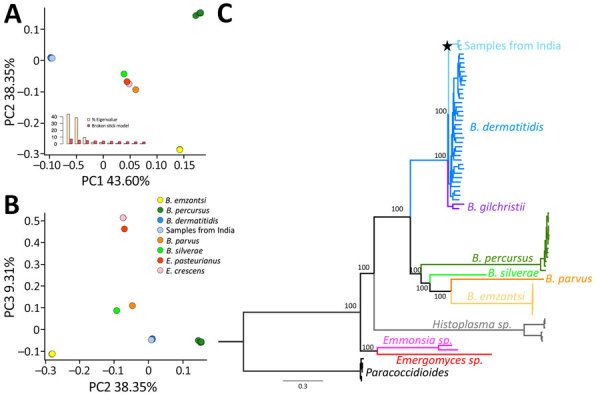
Genetic differentiation between 3 *Blastomyces* *dermatitidis* from India and other dimorphic fungi. A) PC analyses showing the 3 isolates from India (light blue) clustering with *B. dermatitidis* (dark blue) in PC 1 and PC2. The broken stick model (inset) demonstrates the first 3 PCs are significant. B) PC analyses showing the 3 isolates from India (light blue) clustering with *B. dermatitidis *(dark blue) in PC 32 and PC 3. C) Rooted phylogram for autochthonous *B. dermatitidis* isolates from India and the genetic relationships with other *Blastomyces* fungi. A maximum-likelihood tree derived from genomewide concatenated markers using the *B. dermatitidis* reference genome suggests a close phylogenetic relationship between *B. dermatitidis* and the 3 isolates from India. The star shows the node leading to the *B. dermatitidis* lineage from India. Scale bar represents the number of substitutions per site. PC, principal component.

Next, we obtained whole genome sequences for the 3 putative isolates of *Blastomyces* (mean coverage 20.47) (Appendix Table 1). A principal component analysis that included 2 genera from the dimorphic fungi strongly suggested the 3 isolates from India belonged to *Blastomyces* (Figure 2, panel A). Phylogenetic analyses confirmed that the 3 *Blastomyces* isolates from India are closely related to other isolates of *B. dermatitidis* and form a monophyletic group. This group is sister to the North America samples of *B. dermatitidis*. We recovered this topology regardless of the approach to generate the phylogenetic tree (Appendix Figure 1). In all cases, the branch is well supported (ultrafast bootstrap on 1,000 replicates = 100%). Assuming a mutation rate similar to that of other Ascomycetes (*31*,*32*), we found that the origin of the India clade of *B. dermatitidis* is not recent (divergence point estimate ≈0.8 million years, SE 0.12 million years) and might precede the settlement of modern humans in the Indian subcontinent (*33*,*34*).

Finally, we tested whether the monophyletic group of *Blastomyces* isolates from India were differentiated from the other isolates of *B. dermatitidis*. The India group showed the lowest level of diversity of any *Blastomyces* species, lower than that of the North American clade of *B. dermatitidis* and *B. gilchristii* (Table 2). The diversity values are similar in scale to the amount of variability in other fungal species (*35*). The extent of divergence between clades is much higher than π, a proxy of intraclade variability, for both the *B. dermatitidis* and *B. gilchristii* and for the *B. dermatitis* from India versus the rest of *B. dermatitidis* comparisons, suggesting genetic differentiation between the lineage from India and the other lineages of *Blastomyces* (Table 1) (*36*,*37*). Nonetheless, divergent statistics show there is a substantial level of admixture between *B. dermatitidis* from North America and India (Table 2; Appendix Table 3). Those results suggest that, despite the monophyly of this India *Blastomyces* group, the triad of isolates from India still exchanges genetic material with the North America lineage of *B. dermatitidis*.

**Table 2:** Test results on the basis of Patterson’s D statistic showing extensive introgression between the Indian lineage of *Blastomyces dermatitidis* from India and other *Blastomyces* species*

P1	P2	P3	*D*-statistic	F_d_	F4-ratio	Z-score	p value
India	*B. dermatitidis*	*B. gilchristii*	0.0422	0.0515	0.1011	7.1146	5.61 × 10^−13^

*Additional D-statistics values are available in the Appendix Table (https://wwwnc.cdc.gov/EID/article/30/12/24-0830-App1.pdf).

**Table 1:** Approximative 2-sample Fisher-Pitman permutation tests comparing π and DXY values between different lineages of *Blastomyces dermatitidis* from India and North America*

Clade 1	Clade 2	π Clade 1	π Clade 2	D_XY_	Z-score	p value
India	*B. dermatitidis*	0.0003	0.0037	0.0098	21.6158	<0.0001
India	*B. gilchristii*	0.0003	0.002	0.0111	2.9833	0.0046
India	*B. emzantsi*	0.0003	0.0003	0.1920	7.3485	<0.0001
India	*B. parvus*	0.0003	0.2167	0.1908	2.1673	0.0250
India	*B. percursus*	0.0003	0.0056	0.1916	11.6084	<0.001
*B. dermatitidis*	*B. gilchristii*	0.0037	0.0022	0.0127	21.7077	<0.001
*B. dermatitidis*	*B. emzantsi*	0.0037	0.0003	0.1935	29.3221	<0.001
*B. dermatitidis*	*B. parvus*	0.0037	0.2167	0.1926	24.4538	<0.001
*B. dermatitidis*	*B. percursus*	0.0037	0.0056	0.1935	33.565	<0.001

*The pairwise comparison between intraclade variation and the pairwise differentiation suggests divergence between the India clade and the North America clade of *B. dermatitidis*. The nonsignificant value between India and *B. parvus* is an artifact of the paraphyletic nature of *B. parvus*, which has an extremely high π value (Figure). DXY, extent of divergence between clades

## Discussion

In this article, we report a small cluster of cases of blastomycosis in patients from India with no travel history outside the country. By using genomewide sequencing, we determined that the 3 isolates are *B. dermatitidis*. To date, most cases of *B. dermatitidis*–caused blastomycosis outside of North America have had travel history to that continent (*25*). Because of the lack of evidence of person-to-person transmission in blastomycosis, the most likely explanation is that those cases were acquired from the environment. This hypothesis is consistent with environmental collections of *B. dermatitidis* from bat specimens in India (*38*,*39*).

Previous efforts have reported the occurrence of clinical cases of blastomycosis in India, and in all cases, the fungus was assumed to be *B. dermatitidis*; however, those reports preceded the description of other *Blastomyces* species and thus should be revisited. The first report of *Blastomyces* in a patient from India (*40*,*41*) revealed that 3 of 4 suspected cases had a positive immunodiffusion response against anti–*B. dermatitidis* serum precipitins. The efficacy of immunodiffusion to differentiate among *Blastomyces* species is unknown. Because the first report of *Blastomyces* in a patient from India predates DNA sequencing (*40*,*41*), it is unclear whether the samples included isolates from *Blastomyces* species other than *B. dermatitidis*. Previous reports from India have documented isolation of *B. dermatitidis* from insectivorous bats (*38*,*39*,*41*–*43*), and dogs (*44*), suggesting that the disease has an endemic niche in India. A second effort to characterize the global diversity of *Blastomyces* used restriction fragment-length polymorphisms to typify 52 isolates, including 3 isolates from India (*45*). That survey found 2 isolates from India (1 from a patient and 1 from a bat) were genetically similar to each other but were dissimilar from all other isolates included (*45*). Our phylogenetic reconstruction reveals a similar result in that the isolates from India reported in this article form a distinct genetic group within *B. dermatitidis*. 

India has reported cases of blastomycosis imported from the United States (*22*,*41*). In those instances, the fungus was confirmed to be *B. dermatitidis* by DNA sequencing of 2 loci. Our phylogenetic analyses suggest that the lineage of *B. dermatitidis* we sampled in India diverged long ago from the North America lineage. Our results indicate that systematic collections of environmental and patient samples of *Blastomyces*, along with the use of genomics and phylogenetics, are needed to elucidate the natural occurrence and epidemiology of the etiologic agents of blastomycosis. Clinicians should be aware of the possibility of autochthonous blastomycosis in India, particularly among patients with chronic respiratory diseases.

Appendix Additional information about autochthonous *Blastomyces dermatitidis*, India.
