# Supplementary material for: Autochthonous Blastomyces dermatitidis, India
Source: Emerg Infect Dis. 2024 Dec;30(12):2577–82. doi: 10.3201/eid3012.240830 (PMC11616655; doi:10.3201/eid3012.240830)
Supplement: Appendix — Additional information about autochthonous Blastomyces dermatitidis, India. [file 24-0830-Techapp-s1.pdf]

Article DOI: https://doi.org/10.3201/eid3012.240830

EID cannot ensure accessibility for supplementary materials supplied by authors. Readers who have difficulty accessing supplementary content should contact the authors for assistance.

Autochthonous *Blastomyces dermatitidis*, India

Appendix.

Methods

DNA Extraction

We collected ~ 20 mg of mycelia from the cultures on SDA and then grounded with liquid nitrogen and DNA extraction buffer (0.2 mol/L Tris-HCl, 10 mmol/L EDTA, 0.5 mol/L NaCl, 1% SDS) in a mortar and pestle, followed by the phenol, chloroform, and isoamyl alcohol (25:24:1) extraction and ethanol precipitation. We quantified the DNA amount on each extraction using a QUBIT 3 Fluorometer and a dS DNA HS Dye. We used ~100 ng of DNA to prepare libraries using the Truseq Nano library preparation kit (Illumina #20015964) and evaluated library quality in two different ways. We measured the amount of vector and DNA using a Qubit 4.0 fluorometer (Thermofisher #Q33238) and a DNA HS assay kit (Thermofisher #Q32851).

Variant Calling

We mapped reads in the FASTQ files to the *B. dermatitidis* reference genome (GCA_000151595.1; 1) using BWA version 0.7.15 (*2*). We soft-clipped the reads, sorted the resulting BAM files, and marked and filtered the duplicate reads using Samtools version 1.11 (3). We called Single Nucleotide Polymorphisms (SNPs) using the GATK version 4.1.7.0 *HaplotypeCaller* function (*4*,*5*); we set the -ploidy option as 1. We merged the resulting g.vcf files using GATK GenomicsDBImport function and subsequently jointly genotyped the database with the GATK *GenotypeGVCFs* function. For all further analyses, we used only biallelic SNPs and restricted the dataset to sites that fulfilled the passed the following filters: QD <2.0 || FS >60.0 || MQ <30.0 || MQRankSum <-12.5 || ReadPosRankSum <-8.0.

Principal Component Analysis

To determine whether the genetic variation in the Indian isolates of *Blastomyces* was distinct from that found in other lineages, we used Principal Component Analysis (PCA). This approach has the advantage of visualizing how genetic diversity is partitioned in a set of genetic samples. We generated a *beagle* file with ANGSD (*6*) using the bam files described in the section immediately above, filtering sites with more than 20% missing data, a mapping quality lower than 30, and a base quality lower than 20. We then estimated the individual allele frequencies and computed the covariance matrix with *PCAngsd* (function -admix and -selection; 7). We used the function *eigen* in R (*8*) to decompose the covariant matrix into eigenvalues and eigenvectors. Since the combination of the first two principal components (PCs) explained the majority of the variance (See results), we only show those two PCs.

Phylogenetic Tree

To study the genetic relationships of the three isolates, we generated phylogenetic trees using whole genome variation. We used the VCF biallelic variants (described above) to build a phylogenetic tree that included all six sequenced species of *Blastomyces* and the three Indian *Blastomyces*-like isolates. We converted the multisample VCF, generated above, into a concatenated genome-wide alignment in Phylip format using the Python script *vcf2phylip* (*9*). We extracted the 100 kb windows, concatenated them using *bcftools* (*3*), and converted each alignment into Phylip format as above. We then built a ML tree from the genome-wide alignment using IQ-TREE 2 (*10*). We estimated branch support using 1,000 replicates of ultrafast bootstrap approximation (*11*,*12*). We used TreeView (*13*,*14*) to visualize the resulting trees.

Genetic Differentiation

We studied whether the Indian *Blastomyces*-like isolates were differentiated enough to be considered a different phylogenetic species. We calculated the magnitude of genetic differentiation between the Indian isolates and other monophyletic clades identified in the tree (described above, See Results) following Matute and Sepulveda (*15*). In cases of advanced speciation, the mean genetic distance between individuals from different lineages (D_{xy}) is significantly larger than the mean genetic distance between individuals within lineages (nucleotide diversity, π; 15,16) indicating extensive genetic differentiation. We used *Pixy* (*17*) to

calculate D_{xy} and π in all possible pairwise comparisons. *Pixy*'s algorithm includes genotyped invariant sites in the dataset and accounts for missing data to calculate the degree of polymorphism and genetic differentiation. To compare π and D_{xy} values for a given pair of lineages, we used an Approximative Two-Sample Fisher-Pitman Permutation Test (R function *oneway_test*, library *coin*; 18,19) with 1,000 subsampling iterations to determine whether the π values within each of the species in a pair differed from the D_{xy} values.

Divergence Time

We used the phylogenetic reconstruction described above ('Phylogenetic tree') to determine the approximate age of the Indian lineage of *Blastomyces* (See Results). Since there is no fossil record for any of the species of the fungi in the Onygenales order, we relied on the tree branch-lengths to calculate an approximate divergence time. First, we generate an ultrametric tree using the function force.ultrametric (library *phytools*; 20,21). We used the distribution of tree branch lengths and converted them to approximate times using the previously estimated time of divergence between the genus *Blastomyces* and *Histoplasma* (~30 million years; 22,23). Please note this calculation assumes constant mutation and substitution rates across Onygenales, and is meant to give an approximate range of ages and not an exact age of divergence.

Gene Flow

Finally, we studied the extent of gene exchange between the Indian lineage and other *Blastomyces* clades. Instances of advanced speciation show little evidence of gene exchange (15, e.g., 24). We used two variants of the Patterson's *D* statistic (*25–27*) to estimate the amount of introgression in the *Blastomyces* lineage. First, we calculated *D* using *Dtrios* from *Dsuite* (*28*). This metric examines the evidence for introgression for each pair of species in a phylogeny using species quartets and the relative frequency (*p*) of ABBA-BABA sites. To assess whether the *D* statistics deviated from the expectation of no gene flow ($D = 0$), we used the associated Z-value to assess for significance. In *Dtrios*, we provided the genome wide ML tree as the species tree and calculated Patterson's D using *Histoplasma mississippiense* WU24 as the outgroup. Second, we used the program *DInvestigate* from *Dsuite* to calculate the approximate proportion of admixture along the genome with the metric f_d (*29*). Our focus was to assess if there is evidence of introgression between Indian and North American *B. dermatitidis,* but we calculated these two metrics for all possible trios following the recommendations in (*30*).

References

1. Muñoz JF, Gauthier GM, Desjardins CA, Gallo JE, Holder J, Sullivan TD, et al. The dynamic genome and transcriptome of the human fungal pathogen *Blastomyces* and close relative *Emmonsia.* PLoS Genet. 2015;11:e1005493. PubMed https://doi.org/10.1371/journal.pgen.1005493

2. Li H, Durbin R. Fast and accurate short read alignment with Burrows-Wheeler transform. Bioinformatics. 2009;25:1754–60. PubMed https://doi.org/10.1093/bioinformatics/btp324

3. Li H, Handsaker B, Wysoker A, Fennell T, Ruan J, Homer N, et al.; 1000 Genome Project Data Processing Subgroup. The sequence alignment/map format and SAMtools. Bioinformatics. 2009;25:2078–9. PubMed https://doi.org/10.1093/bioinformatics/btp352

4. McKenna A, Hanna M, Banks E, Sivachenko A, Cibulskis K, Kernytsky A, et al. The Genome Analysis Toolkit: a MapReduce framework for analyzing next-generation DNA sequencing data. Genome Res. 2010;20:1297–303. PubMed https://doi.org/10.1101/gr.107524.110

5. Van der Auwera GA, Carneiro MO, Hartl C, Poplin R, Del Angel G, Levy-Moonshine A, et al. From FastQ data to high confidence variant calls: the genome analysis toolkit best practices pipeline. Curr Protoc Bioinforma. 2013;43(1110):11.10.1–11.10.33. **PMID: 2541634**

6. Korneliussen TS, Albrechtsen A, Nielsen R. ANGSD: analysis of next generation sequencing data. BMC Bioinformatics. 2014;15:356. PubMed https://doi.org/10.1186/s12859-014-0356-4

7. Meisner J, Albrechtsen A. Inferring population structure and admixture proportions in low-depth NGS data. Genetics. 2018;210:719–31. PubMed https://doi.org/10.1534/genetics.118.301336

8. R Core Team Rf. R: A language and environment for statistical computing. R foundation for statistical computing Vienna, Austria; 2018. [cited 2019 Jan 15]. https://www.R-project.org

9. Ortiz EM. vcf2phylip v2. 0: convert a VCF matrix into several matrix formats for phylogenetic analysis. [cited 2019 Jan 15]. https://zenodo.org/records/2540861

10. Minh BQ, Schmidt HA, Chernomor O, Schrempf D, Woodhams MD, von Haeseler A, et al. IQ-TREE 2: new models and efficient methods for phylogenetic inference in the genomic era. Mol Biol Evol. 2020;37:1530–4. PubMed https://doi.org/10.1093/molbev/msaa015

11. Hoang DT, Chernomor O, von Haeseler A, Minh BQ, Vinh LS. UFBoot2: improving the ultrafast bootstrap approximation. Mol Biol Evol. 2018;35:518–22. PubMed https://doi.org/10.1093/molbev/msx281

12. Rempel A, Wittler R. SANS serif: alignment-free, whole-genome-based phylogenetic reconstruction. Bioinformatics. 2021;37:4868–70. PubMed https://doi.org/10.1093/bioinformatics/btab444

13. Page RD. TreeView: an application to display phylogenetic trees on personal computers. Comput Appl Biosci. 1996;12:357–8. PubMed

14. Page RD. Visualizing phylogenetic trees using TreeView. Curr Protoc Bioinformatics. 2002:Chapter 6:Unit 6.2

15. Matute DR, Sepúlveda VE. Fungal species boundaries in the genomics era. Fungal Genet Biol. 2019;131:103249. PubMed https://doi.org/10.1016/j.fgb.2019.103249

16. Birky CW Jr. Species detection and identification in sexual organisms using population genetic theory and DNA sequences. PLoS One. 2013;8:e52544. PubMed https://doi.org/10.1371/journal.pone.0052544

17. Korunes KL, Samuk K. pixy: Unbiased estimation of nucleotide diversity and divergence in the presence of missing data. Mol Ecol Resour. 2021;21:1359–68. PubMed https://doi.org/10.1111/1755-0998.13326

18. Zeileis A, Hothorn T, Hornik K. Model-based recursive partitioning. J Comput Graph Stat. 2008;17:492–514. https://doi.org/10.1198/106186008X319331

19. Hothorn T, Hornik K, van de Wiel MA, Zeileis A. Implementing a class of permutation tests: the coin package. J Stat Softw. 2008;28:1–23. https://doi.org/10.18637/jss.v028.i08

20. Revell LJ. phytools: an R package for phylogenetic comparative biology (and other things). Methods Ecol Evol. 2012;3:217–23. https://doi.org/10.1111/j.2041-210X.2011.00169.x

21. Revell LJ. Package 'phytools.' [cited 2019 Jan 15]. https://cran.r-project.org/web/packages/phytools/index.html

22. Kasuga T, White TJ, Koenig G, McEwen J, Restrepo A, Castañeda E, et al. Phylogeography of the fungal pathogen *Histoplasma capsulatum.* Mol Ecol. 2003;12:3383–401. PubMed https://doi.org/10.1046/j.1365-294X.2003.01995.x

23. Sharpton TJ, Stajich JE, Rounsley SD, Gardner MJ, Wortman JR, Jordar VS, et al. Comparative genomic analyses of the human fungal pathogens *Coccidioides* and their relatives. Genome Res. 2009;19:1722–31. PubMed https://doi.org/10.1101/gr.087551.108

24. Mavengere H, Mattox K, Teixeira MM, Sepúlveda VE, Gomez OM, Hernandez O, et al. Paracoccidioides genomes reflect high levels of species divergence and little interspecific gene flow. MBio. 2020;11:e01999–20. PubMed https://doi.org/10.1128/mBio.01999-20

25. Green RE, Krause J, Briggs AW, Maricic T, Stenzel U, Kircher M, et al. A draft sequence of the Neandertal genome. Science. 2010;328:710–22. PubMed https://doi.org/10.1126/science.1188021

26. Durand EY, Patterson N, Reich D, Slatkin M. Testing for ancient admixture between closely related populations. Mol Biol Evol. 2011;28:2239–52. PubMed https://doi.org/10.1093/molbev/msr048

27. Patterson N, Moorjani P, Luo Y, Mallick S, Rohland N, Zhan Y, et al. Ancient admixture in human history. Genetics. 2012;192:1065–93. PubMed https://doi.org/10.1534/genetics.112.145037

28. Malinsky M, Matschiner M, Svardal H. Dsuite - Fast D-statistics and related admixture evidence from VCF files. Mol Ecol Resour. 2021;21:584–95. PubMed https://doi.org/10.1111/1755-0998.13265

29. Martin SH, Davey JW, Jiggins CD. Evaluating the use of ABBA-BABA statistics to locate introgressed loci. Mol Biol Evol. 2015;32:244–57. PubMed https://doi.org/10.1093/molbev/msu269

30. Dagilis AJ, Peede D, Coughlan JM, Jofre GI, D'Agostino ERR, Mavengere H, et al. A need for standardized reporting of introgression: Insights from studies across eukaryotes. Evol Lett. 2022;6:344–57. PubMed https://doi.org/10.1002/evl3.294

31. Jofre GI, Singh A, Mavengere H, Sundar G, D'Agostino E, Chowdhary A, et al. An Indian lineage of *Histoplasma* with strong signatures of differentiation and selection. Fungal Genet Biol. 2022;158:103654. PubMed https://doi.org/10.1016/j.fgb.2021.103654

32. Sepúlveda VE, Márquez R, Turissini DA, Goldman WE, Matute DR. Genome sequences reveal cryptic speciation in the human pathogen *Histoplasma capsulatum*. MBio. 2017;8:e01339–17. PubMed https://doi.org/10.1128/mBio.01339-17

33. Carignan A, Boudhrioua C, Moreira S, Pelletier AA, Dufour K, Pépin J, et al. Changing Patterns of Disease Severity in *Blastomyces dermatitidis* Infection, Quebec, Canada. Emerg Infect Dis. 2021;27:2810–7. PubMed https://doi.org/10.3201/eid2711.210552

34. Maphanga TG, Birkhead M, Muñoz JF, Allam M, Zulu TG, Cuomo CA, et al. Human blastomycosis in South Africa caused by *Blastomyces percursus* and *Blastomyces emzantsi* sp. nov., 1967–2014. J Clin Microbiol. 2020;58:e01661–19. PubMed https://doi.org/10.1128/JCM.01661-19

Appendix Table 1. Patient characteristics of the three *Blastomyces dermatitidis* isolates reported in this study.

Age	Sex	Type of sample
34	Male	Lung aspirate
38	Male	Lymph node biopsy
47	Male	Discharge sinus of the sternum

Appendix Table 2. Sequencing depth and the SRA numbers for each isolate.

SRA/Isolate	Clade	N sites	Mean depth	Reference
109_P_06_S4	*Blastomyces dermatitidis* from India	12560769	23.7254	This Study
143_P_08_S8	*Blastomyces dermatitidis* from India	12561289	15.8974	This Study
WGS_S13	*Blastomyces dermatitidis* from India	12558849	19.1276	This Study
PRJNA178252	*Emmonsia crescens*	12576918	15.2914	(*1*)
PRJNA178252	*Emmonsia crescens*	12576825	24.4543	(*1*)
PRJNA178178	*Blastomyces parvus*	12579103	47.5966	(*1*)
PRJNA178178	*Blastomyces parvus*	12575684	23.0958	(*1*)
PRJNA416769	*Emergomyces pasteurianus*	12577451	17.1335	(*1*)
SRR4024750	*P. restrepiensis*	12577452	5.41929	(*24*)
SRR4024748	*P. restrepiensis*	12577524	5.16314	(*24*)
SAMN05171529	*P. brasiliensis*	12575301	4.15779	(*24*)
SRR9736751	*P. venezuelensis*	12575361	3.54549	(*24*)
SRR9736752	*P. venezuelensis*	12573112	2.09213	(*24*)
SRR9736753	*P. venezuelensis*	12576272	4.90977	(*24*)
104_p_06_S19	*Histoplasma*, Indian clade	12576785	9.09513	(*31*)
104_P_19_S5	*Histoplasma*, Indian clade	12577399	11.3325	(*31*)
107_P_06_S1	*Histoplasma*, Indian clade	12572108	1.96497	(*31*)
SRX3350830	*H. mississippiense*	12579690	5.62048	(*32*)
SRX3350838	*H. mississippiense*	12576784	10.1362	(*32*)
SRX3350840	*H. mississippiense*	12576661	10.2807	(*32*)
SRR15390220	*Blastomyces dermatitidis*	12547490	14.8293	(*33*)
SRR15390222	*Blastomyces dermatitidis*	12546929	23.3171	(*33*)
SRR15390225	*Blastomyces dermatitidis*	12554966	18.25	(*33*)
SRR15390227	*Blastomyces dermatitidis*	12546055	16.5328	(*33*)
SRR15390235	*Blastomyces dermatitidis*	12553886	15.6108	(*33*)
SRR15390237	*Blastomyces dermatitidis*	12560467	19.4161	(*33*)
SRR15390240	*Blastomyces dermatitidis*	12553251	10.4586	(*33*)
SRR15390246	*Blastomyces dermatitidis*	12548401	13.8083	(*33*)
SRR15390247	*Blastomyces dermatitidis*	12550774	23.1604	(*33*)
SRR15390251	*Blastomyces dermatitidis*	12549155	15.8052	(*33*)
SRR15390252	*Blastomyces dermatitidis*	12550967	19.3192	(*33*)
SRR15390260	*Blastomyces dermatitidis*	12551151	17.9408	(*33*)
SRR15390262	*Blastomyces dermatitidis*	12552518	23.1796	(*33*)
SRR15390269	*Blastomyces dermatitidis*	12541869	14.8117	(*33*)
SRR15390272	*Blastomyces dermatitidis*	12548867	21.0119	(*33*)
SRR15390277	*Blastomyces dermatitidis*	12542546	20.4215	(*33*)
SRR15390285	*Blastomyces dermatitidis*	12548382	19.4143	(*33*)
SRR15390287	*Blastomyces dermatitidis*	12536074	10.9878	(*33*)
SRR15390290	*Blastomyces dermatitidis*	12547352	26.7468	(*33*)
SRR15390291	*Blastomyces dermatitidis*	12541545	16.723	(*33*)
SRR15390294	*Blastomyces dermatitidis*	12559299	15.7778	(*33*)
SRR15390303	*Blastomyces dermatitidis*	12547910	24.073	(*33*)
SRR15390305	*Blastomyces dermatitidis*	12551862	16.589	(*33*)
SRR15390306	*Blastomyces dermatitidis*	12550367	24.1023	(*33*)
SRR15390308	*Blastomyces dermatitidis*	12519241	19.0574	(*33*)
SRR15390309	*Blastomyces dermatitidis*	12545281	17.5147	(*33*)
SRR15390314	*Blastomyces dermatitidis*	12554583	22.2366	(*33*)
SRR15390319	*Blastomyces dermatitidis*	12552928	21.0374	(*33*)
SRR15390320	*Blastomyces dermatitidis*	12552870	21.4327	(*33*)
SRR15390326	*Blastomyces dermatitidis*	12548642	20.143	(*33*)
SRR15390330	*Blastomyces dermatitidis*	12543867	12.4004	(*33*)
SRR15390332	*Blastomyces dermatitidis*	12537202	12.9073	(*33*)
SRR15390346	*Blastomyces dermatitidis*	12549203	11.0397	(*33*)
SRR15390347	*Blastomyces dermatitidis*	12545572	18.4175	(*33*)
SRR15390348	*Blastomyces dermatitidis*	12550345	17.5644	(*33*)
SRR11849826	*Blastomyces gilchristii*	12567842	20.4578	NA
SRR10992698	*Blastomyces percursus*	12577737	19.6691	NA
SRR10992699	*Blastomyces percursus*	12578192	35.5259	NA
SRR10992700	*Blastomyces percursus*	12578008	26.3424	NA
SRR10992701	*Blastomyces percursus*	12578320	32.7573	NA

SRA/Isolate	Clade	N sites	Mean depth	Reference
QGQM01000001.1	*Blastomyces percursus_5*	12575207	24.5276	(*34*)
QGQO01000001.1	*Blastomyces percursus_6*	12575556	34.3875	(*34*)
QGQF01000001.1	*Blastomyces emzantsi_1*	12576458	41.7562	(*34*)
QGQG01000001.1	*Blastomyces emzantsi_2*	12576389	49.3843	(*34*)
QGQH01000001.1	*Blastomyces emzantsi_3*	12576399	42.3668	(*34*)
QGQE01000001.1	*Blastomyces emzantsi_4*	12575932	44.316	(*34*)
QGQJ01000001.1	*Blastomyces emzantsi_5*	12575287	29.2844	(*34*)
QKWI01000001.1	*Blastomyces emzantsi_7*	12575131	31.0548	(*34*)
QGQK01000001.1	*Blastomyces emzantsi_6*	12575651	34.7016	(*34*)
QGQP01000001.1	*Blastomyces percursus_8*	12576101	29.4733	(*34*)
QGQQ01000001.1	*Blastomyces percursus_9*	12575355	26.1185	(*34*)
QGQI01000001.1	*Blastomyces percursus_7*	12575125	22.7218	(*34*)
QGQS01000001.1	*Blastomyces percursus_10*	12573899	21.2033	(*34*)
QGQL01000001.1	*Blastomyces emzantsi_9*	12575769	38.6096	(*34*)
QGQT01000001.1	*Blastomyces percursus_11*	12564998	4.59623	(*34*)
QGQN01000001.1	*Blastomyces percursus_12*	12575733	28.9503	(*34*)
QGQR01000001.1	*Blastomyces percursus_13*	12575857	28.697	(*34*)
PRJNA450721	*Blastomyces percursus_14*	12574504	33.9987	(*34*)

Appendix Table 3. D-statistic values for all triads in the genus *Blastomyces*, including the Indian clade of *B. dermatitidis*.

P1	P2	P3	D-statistic	z-score	p-value	F4-ratio
India	*B. dermatitidis*	*B. gilchristii*	0.0422	7.1146	0.0000	0.1010
B. gilchristii	*B. dermatitidis*	*B. emzantsi*	0.2980	22.8753	0.0000	0.2117
B. gilchristii	*B. dermatitidis*	*B. percursus*	0.2904	25.6929	0.0000	0.2334
B. dermatitidis	India	*B. emzantsi*	0.0217	3.5927	0.0002	0.0283
B. dermatitidis	India	*B. percursus*	0.0155	2.6263	0.0043	0.0234
B. percursus	*B. emzantsi*	*B. dermatitidis*	0.0018	0.7833	0.2167	0.0014
B. gilchristii	India	*B. emzantsi*	0.3091	24.2820	0.0000	0.2341
B. gilchristii	India	*B. percursus*	0.2947	24.8340	0.0000	0.2515
B. emzantsi	*B. percursus*	*B. gilchristii*	0.0092	2.8301	0.0023	0.0037
B. percursus	*B. emzantsi*	India	0.0073	3.1616	0.0008	0.0050
